# Safety and efficacy of iodine-125 seed strand for intraluminal brachytherapy on ureteral carcinoma

**DOI:** 10.3389/fonc.2023.1081258

**Published:** 2023-03-27

**Authors:** Yonghua Bi, Dechao Jiao, Jianhao Zhang, Jianzhuang Ren, Xinwei Han, Kefeng Guo, Xueliang Tu

**Affiliations:** ^1^ Department of Interventional Radiology, The First ffiliated Hospital of Zhengzhou University, Zhengzhou, China; ^2^ Department of Oncology, Yellow River Sanmenxia Affliated Hospital of Henan University of Science and Technology, Sanmenxia, China; ^3^ Department of Clinical Laboratory, Yellow River Sanmenxia Affliated Hospital of Henan University of Science and Technology, Sanmenxia, China

**Keywords:** iodine-125 seed strand, intraluminal brachytherapy, ureteral carcinoma, nephrostomy, renal pelvis

## Abstract

**Objective:**

Our aim is to evaluate the safety and efficacy of iodine-125 seed strand for intraluminal brachytherapy on ureteral carcinoma.

**Methods:**

From November 2014 to November 2021, 22 patients with ureteral cancer not suitable for surgical resection were enrolled. Iodine-125 seed strand was inserted under c-arm CT and fluoroscopic guidance. The technical success rate, complications, disease control rate, and survival time were evaluated. Hydronephrosis Girignon grade and ureteral cancer sizes before and after treatment were compared.

**Results:**

A total of 46 seed strands were successfully inserted and replaced, with a technical success rate of 100% and median procedure time of 62 min. No procedure-related death, ureteral perforation, infection, or severe bleeding occurred. Minor complications were observed in eight (36.4%) patients, and migration of seed strand was the most common complication. Six months after seed strand brachytherapy, one complete response, three partial responses, and five stable diseases were evaluated, and the disease control rate was 64.3%. The Girignon grade of hydronephrosis was significantly improved 1 to 3 months after seed strand insertion. Disease control rates were 94.4, 62.5, and 64.3% at 1-, 3-, and 6-month follow-up. Twenty patients were successfully followed up, with a mean follow-up of 18.0 ± 14.5 months. The median overall survival and progress-free survival were 24.7 and 13.0 months, respectively.

**Conclusion:**

Iodine-125 seed strand is safe and effective for intraluminal brachytherapy and can be used as an alternative to patients with ureteral carcinoma who are not suitable for surgical resection or systemic combined therapy.

## Introduction

Ureter transitional cell carcinoma is a relative rare disease, which account for about 1–3% of urinary transitional cell carcinoma cases (1). Surgical resection is the standard treatment for ureteral carcinoma. Radical ureterectomy with excision of the kidney and bladder cuff resection is generally recommended (2), considering that transitional cell carcinoma may develop metachronously or synchronously in a multifocal manner at different urinary tract sites. However, radical resection causes the loss of kidney to patient, which is not an easy choice for patients with renal insufficiency, isolated kidney, or bilateral ureteropathy. Some patients do not undergo radical nephroureterectomy due to advanced age, comorbidities, or intolerance or refusal of the procedure (3).

Traditional external radiotherapy is usually recommended for patients with advanced ureteral carcinoma and may be advantageous in several aspects. For example, adjuvant radiotherapy in the setting of locally advanced ureteral carcinoma improves locoregional control following definitive surgery (4). However, in ureteral lesions often close to intestines, spinal cord, and blood vessels, long-term high-dose radiotherapy may result in intestinal injury, spinal cord damage, or retroperitoneal fibrosis (5). As a novel kind of radiotherapy, intraluminal brachytherapy with radioactive iodine-125 seed strand has been used in some malignant tumors patients who cannot undergo or refuse surgical resection and shows encouraging efficacy (6), including advanced/recurrent esophageal cancer (7), malignant biliary obstruction (8), and hepatocellular carcinoma with portal vein tumor thrombus (9). Inspired by previous intraluminal brachytherapy, we wanted to explore whether iodine-125 seed strand brachytherapy is also suitable for the treatment of malignant ureteral obstruction. We herein presented 22 patients with ureteral carcinoma who were treated with intraluminal brachytherapy by iodine-125 seed strand insertion.

## Materials and methods

### Patients

**Figure 1:**
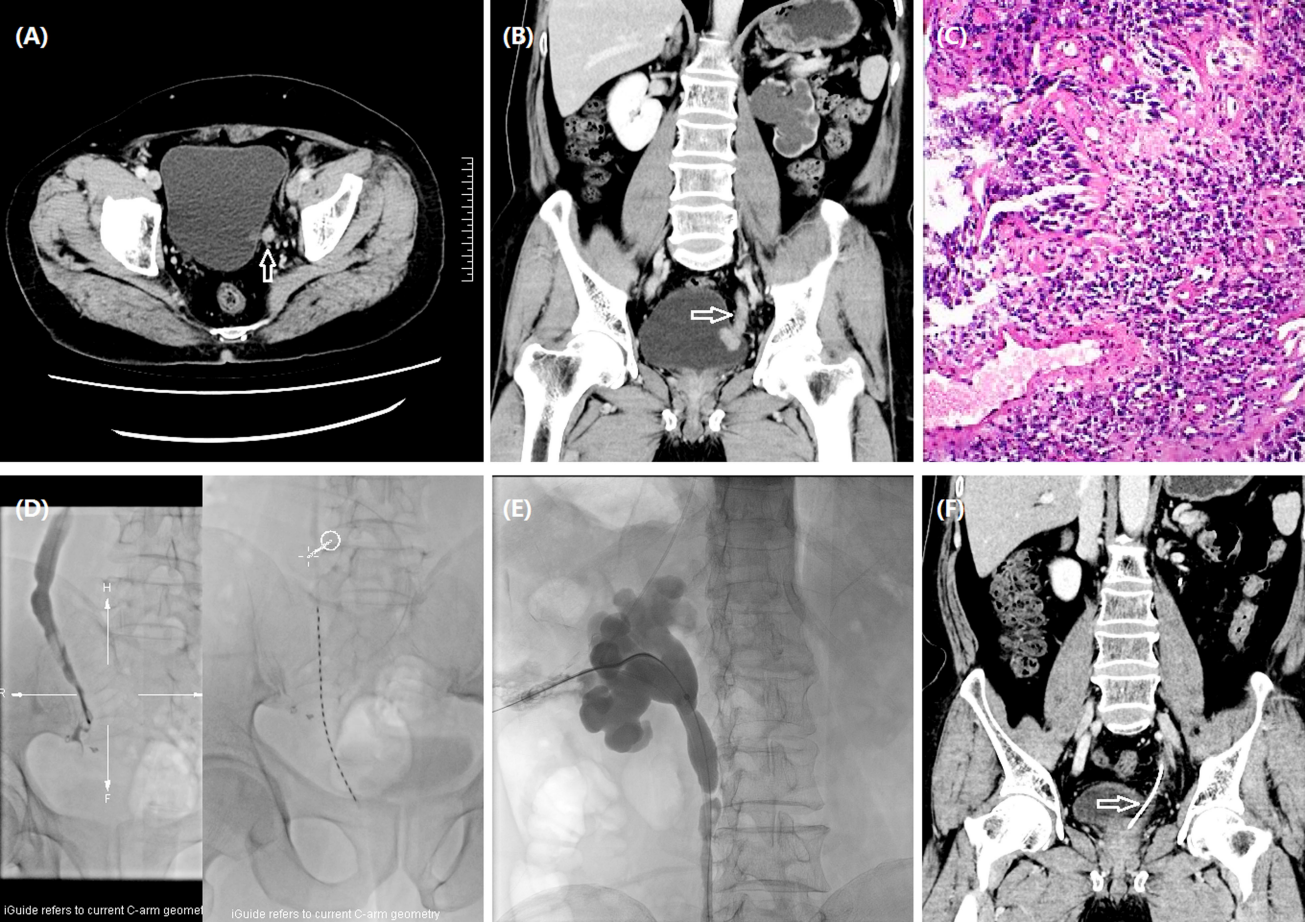
Iodine-125 seed strand placement for a 70-year man with ureteral carcinoma. **(A)** A left ureteral carcinoma (arrow) was shown by CT. **(B)** The tumor (arrow) was located in the left lower ureteric segment and bladder entrance with significantly dilated renal pelvis and thinning of renal cortex. **(C)** Pathology showed low-grade papillary urothelial carcinoma of the ureter without definite infiltration. **(D)** Urothrography revealed a 54.7-mm-long occlusion in the left lower ureter, and an iodine-125 seed strand with 23 seeds was inserted. **(E)** Nephrostomy was performed under fluoroscopic guidance. **(F)** Complete response (arrow) was detected by CT after 5.9 months.

**Figure 2:**
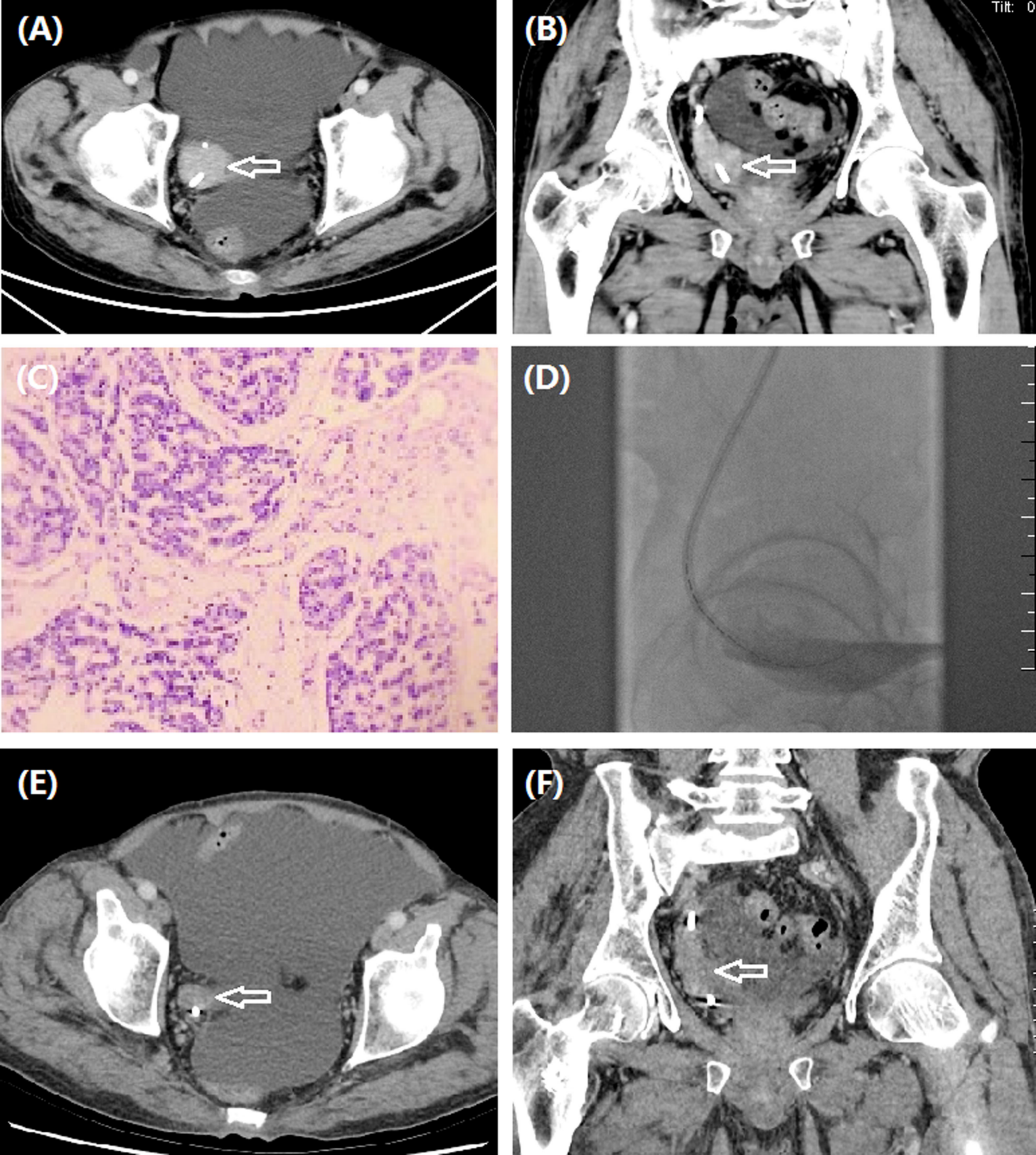
A 61-year man treated by iodine-125 seed strand for right ureteral carcinoma. **(A, B)** CT revealed ureteral carcinoma (arrow) of right lower ureter. **(C)** Pathology was confirmed to be ureteric papillary urothelial carcinoma with tumor invasion in the local lamina propria. **(D)** Iodine-125 seed strand with 15 seeds was inserted in the occlusive segment. **(E–F)** Stable tumor (arrow) was detected by CT after 6.0 months.

**Figure 3:**
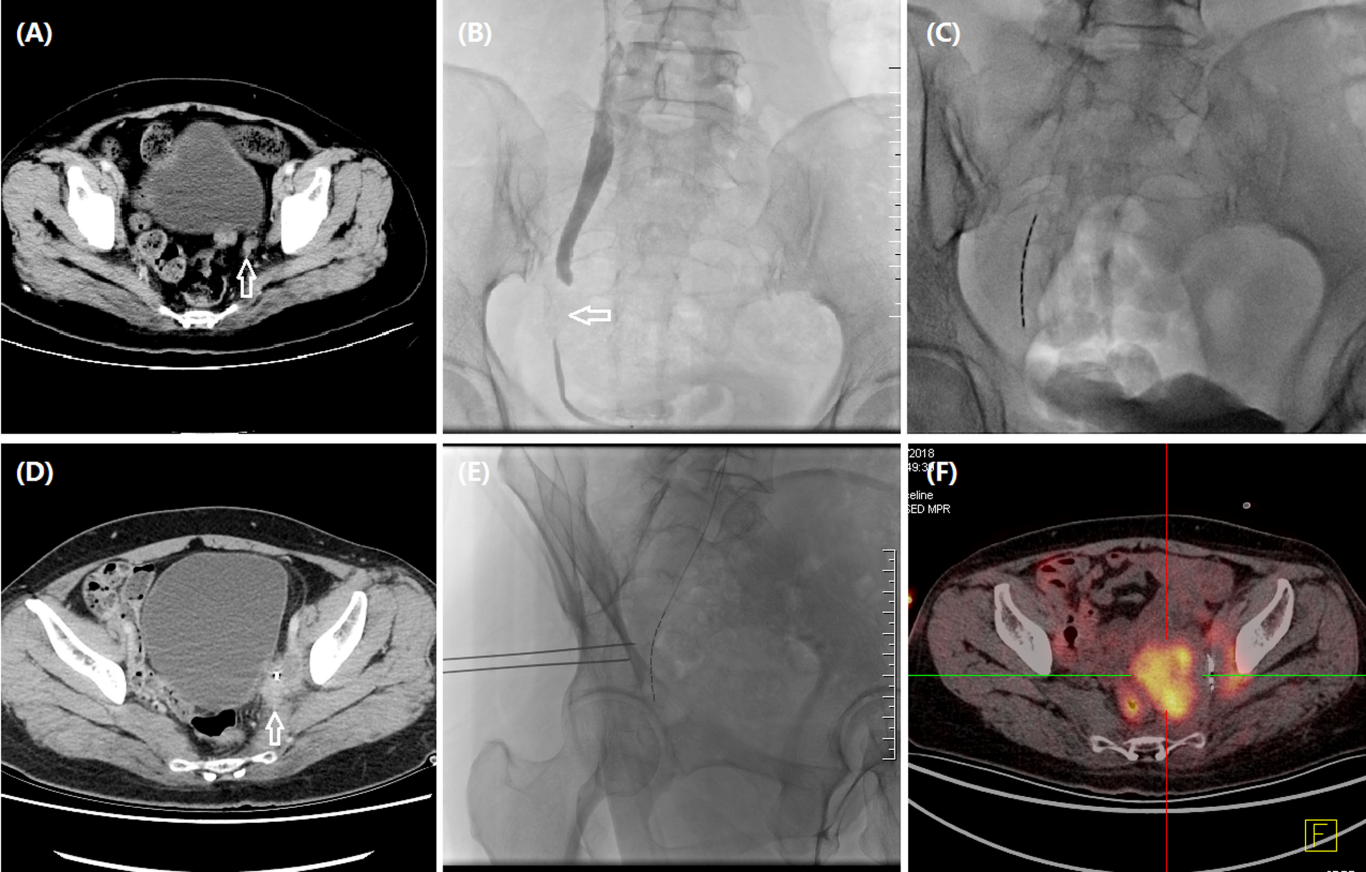
A 48-year woman treated by iodine-125 seed strand for metastatic carcinoma of ureter. **(A)** CT revealed metastatic carcinoma of left ureter due to recurrent cervical carcinoma (arrow). **(B)** Urothrography revealed a segmental occlusion (arrow) in the lower segment of left ureteral segment, approximately 27.2 mm in length. **(C)** Iodine-125 seed strand with 10 seeds was inserted in the occlusive segment. **(D)** CT revealed an enlarged tumor (arrow) 2.2 months later. **(E)** Progressive tumors were treated with percutaneous particle implantation, and a total of 10 particles were implanted. **(F)** Tumor progression was detected by PET-CT after 12.0 months’ follow-up.

From November 2014 to November 2021, all patients with ureteral carcinoma treated by intraluminal brachytherapy were enrolled. Contrast-enhanced computed tomography (CT) and/or magnetic resonance imaging (MRI) were carried out at baseline (**Figures 1A–C**, **2A–C**, **3A**). The inclusion criteria: patients with age > 18 years, with an estimated life expectancy exceeds 3 months; patients with renal insufficiency, isolated kidney, or bilateral ureteropathy; patients not suitable for radical nephroureterectomy or combined therapy (GC chemotherapy + PD-1) due to advanced age, comorbidities; and patients with intolerance or refusal of surgical resection. Exclusion criteria: severe cardiovascular comorbidities including unstable angina, myocardial infarction, or uncontrolled hypertension; coagulopathy or bleeding diathesis; and severe infection. Ethical approval was waived by the Institutional Review Board of University due to its retrospective nature. Informed consent had been obtained from all patients before procedure.

### Nephrostomy under c-arm CT and fluoroscopic guidance

The patient was prone on the examination table, and percutaneous renal pelvis puncture was performed under the virtual navigation of c-arm CT iGuide and fluoroscopic guidance. The upper segment of the renal pelvis and the ureter were visible by urography, and the involved ureter was shown as local stenosis or occlusion. The lesion in the ureter was shown as filling defect of the contrast agent. The ureteral obstruction length was measured to assess the number of seed placements. For severe upper urinary tract obstruction patients, a 0.035 inch guidewire and a 5F catheter were used to carefully pass through the ureteral stenosis or occlusion segment into the bladder. A stiff guidewire was then exchanged, and 8F short sheath was inserted. Another stiff guidewire was introduced *via* the short sheath. After removal of short sheath, an 8.5F drainage tube was inserted along a stiff guidewire into renal pelvis for urine drainage.

### Iodine-125 seed strand

The seed strand was introduced into the lesion segment along another stiff guidewire and fixed to avoid migration. The iodine-125 seeds (seed activity: 0.7 mCi, Saide Biological Technology Co., Ltd., Tianjin, China) are 0.8 mm in diameter and 4.8 mm in length and have a half-life of 59.6 days and an irradiation distance of 17 mm. A seed implantation gun was used to push the seeds into a 3F catheter, arranging the iodine-125 seeds in a row within the catheter to form a seed strand. The length of both strand ends was 2 cm longer than the lesion segment. Both ends of the seed strand are sealed with fire to avoid seed migration. The following formula was used to assess the number (*N*) of seeds implanted: *N* = (lesion segment length + 2 cm + 2 cm)/0.45. In this study, the mean D90 (the dose covering 90% of the gross tumor volume) and the organ at risk (OAR) doses were 50.7 and 3.8 Gy, respectively. The seed strand and the drainage tube were removed or exchanged 2–3 months after the insertion (**Figures 1D, E**, **2D**, **3B, C, E**).

### Evaluation and follow-up

The technical success rate, complications, disease control rate, and survival time were evaluated. Hydronephrosis Girignon grade and ureteral cancer sizes before and after treatment were compared. Technical success rate was defined as the successful placement of nephrostomy and seed strand. Two to 3 months later, abdominal CT scan was performed to evaluate clinical efficacy. Tumor evaluation was performed by using RECIST to evaluate the efficacy (**Figures 1F**, **2E, F**, **3D, F**).

## Results

### Patient characteristics

From November 2014 to November 2021, 22 patients with ureteral cancer not suitable for surgical resection were enrolled in this study, including 12 men and 10 women (mean age 68.1 ± 11.1 years, range 46–84 years). Disease characteristics and baseline demographics are shown in **Table 1**. Local metastasis was present in 10 patients (45.5%), and distant metastasis was found in three patients (13.6%). Twelve patients were confirmed by ureteroscopy or percutaneous forceps biopsy as ureter transitional cell carcinoma. Nine patients (40.9%) complained of gross hematuria, and median duration of symptom was 6.0 months. Five patients (22.7%) received synchronous radiochemotherapy. Five patients with metastatic carcinoma of ureter from cervical cancer (22.7%) received prior radiochemotherapy, including concurrent radiochemotherapy (TP chemotherapy + intracavitary brachytherapy 15Gy/3F) in four patients and DP chemotherapy (Docetaxel + nedaplatin) in the remained one patient.

**Table 1:** Baseline characteristics of enrolled patients.

Variables	Data
Mean age (range), years	68.1 ± 11.1 (46–84)
Course of disease, months	6.0 (1.2, 20.5)
Lesion types	
Advanced ureteral carcinoma	13 (59.1%)
Recurrent ureteral carcinoma	4 (18.2%)
Metastatic carcinoma of ureter	5 (22.7%)
Upper/middle/lower segment	4 (18.2%)/3(13.6%)/15(68.2%)
Synchronous radiochemotherapy	4 (18.2%)
Local metastasis	10 (45.5%)
Distant metastasis	3 (13.6%)
Gross hematuria	9 (40.9%)
Comorbidities	
Diabetes mellitus	6 (27.3%)
Coronary heart disease	3 (13.6%)
Old cerebral infarction	3 (13.6%)
Hypertension	9 (40.9%)

### Procedure outcomes of iodine-125 seed strand

As shown in **Table 2**, a total of 46 seed strand sessions were successfully inserted and replaced in 22 patients, with a mean of 2.1 ± 1.5 sessions. Ten patients (45.5%) completed two to five sessions of seed strand replacement or removal, with median indwelling interval of 3.0 months. The median procedure time was 62 min, and each strand was loaded with 20 iodine-125 seeds. Eighteen patients (81.8%) received other interventional treatments, including transcatheter arterial chemoembolization (*n* = 10) and percutaneous seed implantation (*n* = 3), and both (*n* = 5) for progressive and migrated tumors.

**Table 2:** Clinical data.

Variables	Data
Procedure time, minutes	62 (50.0, 90.0)
Indwelling interval, months	3.0 (1.8, 3.6)
Number of seeds	20 (15.0, 24.5)
Median hospital stay, days	16.0 (8.5, 27.5)
Median medical cost, ×10^4^ Renminbi	4.3 (2.4, 6.1)
Complications, *n* (%)	8 (36.4%)
Migration of seed strand	5 (22.7%)
Mild abdominal pain	3 (13.6%)
Obstruction of tube	1 (4.5%)
Fever	2 (9.1%)
Girignon before/after placement	4.1 ± 0.9/1.9 ± 0.6*
Lesion diameter/length, mm	
Before seed strand procedure	20.0 (15.6–23.9)/35.0 (20.0–51.4)
1 month later	12.0 (12.0–17.6)/14.8 (2.8–28.0)
3 months later	22.0 (14.2–35.0)/27.9 (19.0–43.5)
6 months later	20.0 (17.0–27.0)/26.5 (3.0–40.0)

*p < 0.0001.

### Complications

Minor complications were observed in eight (36.4%) patients; no severe adverse events occurred such as procedure-related death, ureteral perforation, infection, or severe bleeding. Migration of seed strand was the most common complication, which was observed in five patients (22.7%). Seed strand adjustment, replacement, or removal was performed for migrated strand. Mild abdominal pain of renal puncture site was observed in three patients (13.6%), and fever was observed in two patients (9.1%). One patient showed obstruction of nephrostomy tube, and the tube was successfully replaced.

### Tumor response

Six months after seed strand brachytherapy, one complete response, three partial responses and five stable diseases were evaluated, and the disease control rate was 64.3%. The Girignon grade of hydronephrosis was significantly improved 1–3 months after seed strand insertion. One patient achieved a complete response 6 months after seed strand placement according to the RECIST criteria. Partial response was observed in four, five, and three patients at 1-, 3-, and 6-month follow-up. The objective response rates were 22.2, 31.3, and 28.6%, respectively, at 1, 3, and 6 months. The disease control rates were 94.4, 62.5, and 64.3%, respectively at 1, 3, and 6 months (**Table 3**).

**Table 3:** Treatment responses.

Response	1 month	3 months	6 months
Complete response	0 (0.0%)	0 (0.0%)	1 (7.1%)
Partial response	4 (22.2%)	5 (31.3%)	3 (21.4%)
Stable disease	13 (72.2%)	5 (31.3%)	5 (35.7%)
Progressive disease	1 (5.6%)	6 (37.5%)	5 (35.7%)
Objective response rate	4 (22.2%)	5 (31.3%)	4 (28.6%)
Disease control rate	17 (94.4%)	10 (62.5%)	9 (64.3%)

### Survival

**Figure 4:**
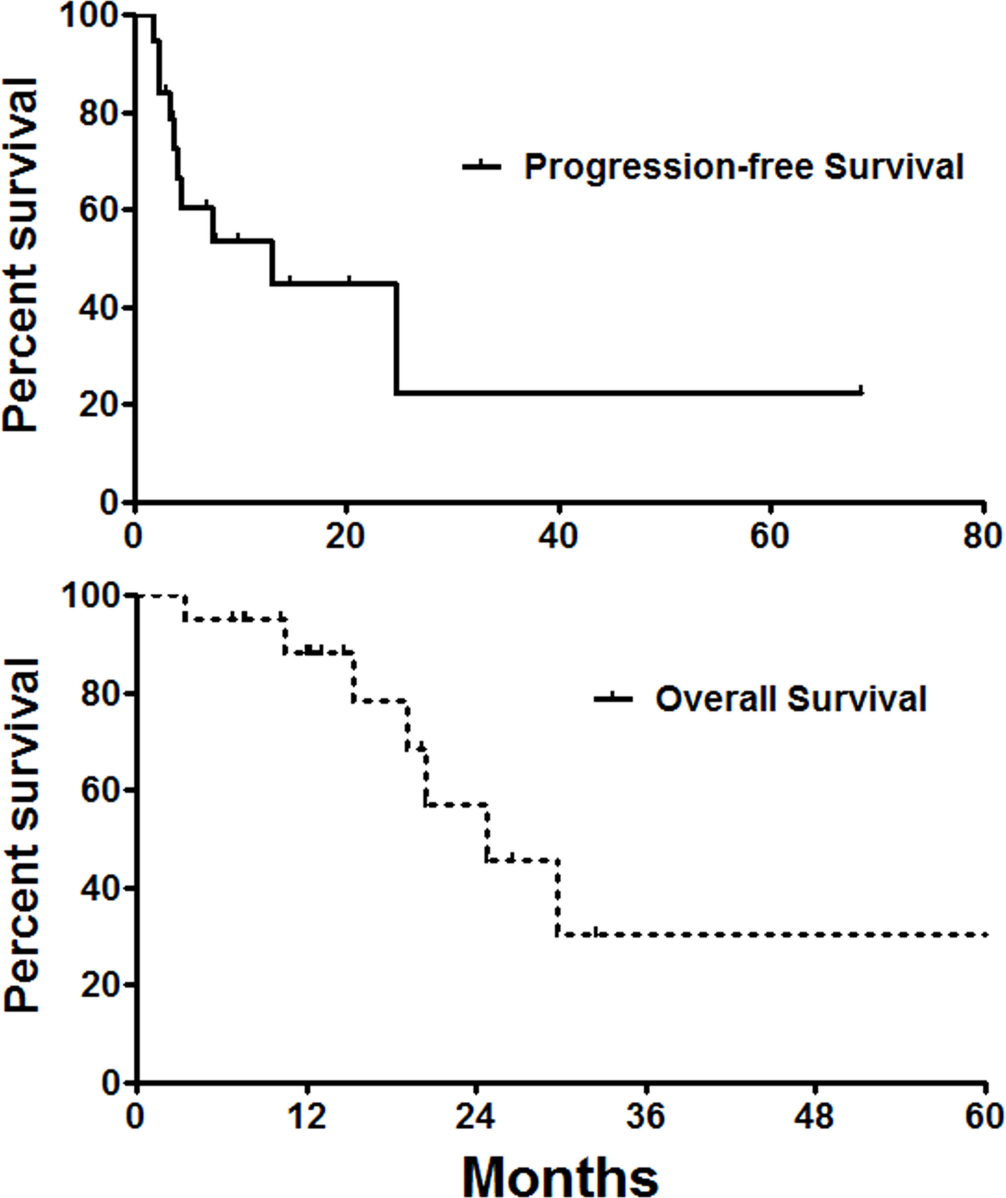
Survival follow-up. The median overall survival and progress-free survival were 24.7 and 13.0 months, respectively.

Two patients were lost of follow-up, with a mean follow-up of 18.0 ± 14.5 months and follow-up rate of 90.9%. Seven patients (31.8%) died of tumor progression. The median overall survival and progress-free survival were 24.7 and 13.0 months, respectively (**Figure 4**). The 6-, 12-, and 36-month overall survival rates were 95.0, 88.2, and 30.5%, respectively. The 6-, 12-, and 36-month progression-free survival rates were 60.5, 44.8, and 22.4%, respectively.

## Discussion

Since the renal pelvis, ureter, and bladder all originate from the mesoderm, with similar biological and morphology behavior, radical resection of bladder and ureteral carcinoma requires simultaneous resection of the bladder and ureter to reduce tumor metastasis and recurrence. A recurrence rate of 15–50% is observed in ureteral carcinoma after surgical resection of unilateral upper urinary carcinoma, and even reported a high rate of up to 70% (2, 10). Radical nephroureterectomy is traumatic and costly, patients have to lose their kidneys, but the recurrence rate is still high. This is not an easy choice for patients with renal insufficiency, isolated kidney, or bilateral ureteropathy. Some patients do not undergo radical nephroureterectomy due to advanced age, comorbidities, or intolerance or refusal of the procedure (3). In addition, locoregional urothelial malignancies of urinary tract was treated by adjuvant radiotherapy or combined with chemotherapy (5, 11, 12), and confocal laser endomicroscopy has been used for upper tract urothelial carcinoma (13, 14), although radiotherapy is rarely used for ureter tumors.

Currently, more minimally invasive alternative methods are urgently needed in clinical practice for such patients with ureteral carcinoma who are not suitable for surgical resection. Minimally invasive treatment using interstitial puncture and the implantation of iodine-125 seeds has become a common clinical technique for the treatment of various solid malignancies (15), such as oral and maxillofacial malignant tumors (16). However, it remains unclear whether malignant tumors of cavity organs such as ureteral carcinoma can be treated by radioactive seeds brachytherapy, although cavity organs such as esophageal cancer have been treated with low-dose-rate brachytherapy with radioactive stent (17) and nutrient tube (7). It has also been reported that iodine-125 seeds loading inside the catheter to form a seed strand structure to treat malignant bile duct obstruction (18, 19) and portal vein tumor thrombus (20) showed satisfactory clinical results.

The ureter is a thin, long, and tubular shape organ, and ureteral carcinoma can easily cause ureteral obstruction and hydrops, even when the tumor is very small. Inspired by previous intraluminal brachytherapy, we aimed to explore whether iodine-125 seed strand brachytherapy is also suitable for the treatment of malignant ureteral obstruction (21). The iodine-125 seeds have an irradiation distance within 1.7 cm; intraluminal low-dose brachytherapy may theoretically play a safer and better role for ureteral carcinoma (21). We herein presented 22 patients with ureteral carcinoma who were treated with intraluminal brachytherapy by iodine-125 seed strand insertion.

In this study, 46 seed strands were successfully inserted and replaced, with a technical success rate of 100%. The Girignon grade of hydronephrosis was significantly improved 1–3 months after seed strand insertion. Disease control rates were 94.4, 62.5, and 64.3% at 1-, 3-, and 6-month follow-up. The median overall survival and progression-free survival were 24.7 and 13.0 months, respectively. Our results suggest that intraluminal brachytherapy by iodine-125 seed strand is feasible and effective alternative to patients who are not suitable for surgical resection.

Regarding safety, it is also reported that iodine-125 brachytherapy may cause radiation damage to vascular (22), nerve (23), and tracheal structures (24), but the results all showed mild target and surrounding organ damage. In our study, no procedure-related death or severe complications occurred. Only eight (36.4%) patients had minor complications, and migration of seed strand was the most common complication.

There were several limitations in this study: (1) Only 22 patients were studied in this retrospective study; further studies with larger sample size are required. (2) Less than half of patients received more than two sessions of seed strand, which may contribute to confounding factor affecting efficacy evaluation. (3) The radiation distance of the seed is short, and it may not be enough for metastatic cancer or large lesions; other local or systematic treatments are required. (4) The combined therapy is recommended for advanced ureteral carcinoma. In this study, patients are not suitable for radical nephroureterectomy or combined therapy (GC chemotherapy + PD-1) due to advanced age, comorbidities. Theoretically, the combination of local iodine-125 strand therapy with the systemic combined therapy is likely to achieve better efficacy for advance ureteral carcinoma.

## Conclusion

As a localized and palliative therapy, iodine-125 seed strand is safe and effective for intraluminal brachytherapy and can be used as an alternative to patients with ureteral carcinoma who are not suitable for surgical resection or systemic combined therapy.

## Data availability statement

The raw data supporting the conclusions of this article will be made available by the authors, without undue reservation.

## Ethics statement

Ethical approval was waived by the Institutional Review Board of University due to its retrospective nature. The patients/participants provided their written informed consent to participate in this study.

## Author contributions

XH and JR conceptualized the study. YB, DJ, and JZ developed the methodology. DJ and JZ validated the study. YB, DJ, and JZ performed the formal analysis. YB and DJ wrote and prepared the original draft. KG and XT wrote, reviewed, and edited the article. XH and JR conducted the visualization. XH and JR supervised the study. All authors contributed to the article and approved the submitted version.
